# Mesentery-Sparing Technique: a New Intracorporeal Approach for Urinary Diversion in Robot-Assisted Radical Cystectomy

**DOI:** 10.1590/S1677-5538.IBJU.2024.0153

**Published:** 2024-05-20

**Authors:** Eliney Ferreira Faria, Carlos Vaz de Melo Maciel, Pablo Almeida Melo, Marcos Tobias-Machado, Roberto Dias Machado, Rodolfo Borges dos Reis, Rodrigo José Costa-Gualberto

**Affiliations:** 1 Departamento de Urologia Hospital Felício Rocho Belo Horizonte MG Brasil Departamento de Urologia, Hospital Felício Rocho, Belo Horizonte, MG, Brasil;; 2 Programa de Pós-Graduação em Ciências da Saúde Faculdade Ciências Médicas de Minas Gerais Belo Horizonte MG Brasil Programa de Pós-Graduação em Ciências da Saúde, Faculdade Ciências Médicas de Minas Gerais – FCM-MG, Belo Horizonte, MG, Brasil;; 3 Departamento de Uro-Oncologia Instituto do Câncer Dr. Arnaldo Vieira de Carvalho São Paulo SP Brasil Departamento de Uro-Oncologia, Instituto do Câncer Dr. Arnaldo Vieira de Carvalho, São Paulo, SP, Brasil;; 4 Departamento de Cirurgia e Urologia Hospital do Câncer de Barretos Barretos SP Brasil Departamento de Cirurgia e Urologia, Hospital do Câncer de Barretos, Barretos, SP, Brasil;; 5 Departamento de Cirurgia e Anatomia Faculdade de Medicina de Ribeirão Preto Universidade de São Paulo Ribeirão Preto SP Brasil Divisão de Urologia, Departamento de Cirurgia e Anatomia, Faculdade de Medicina de Ribeirão Preto, Universidade de São Paulo, Ribeirão Preto, SP, Brasil

**Keywords:** Robotic Surgical Procedures, Urinary Bladder Neoplasms, Urinary Diversion

## Abstract

**Background:**

Robotic-assisted radical cystectomy (RARC) with intracorporeal urinary diversion (ICUD) is associated with significant morbidity and mortality. We present an alternative technique that preserves the complete mesenteric vascularization during the isolation of the intestinal segment used in ICUD, including distal vessels. This approach aims to minimize the risk of ischemia in both the ileal anastomosis and the isolated loop at the diversion site.

**Methods:**

This cohort study included 31 patients, both male and female, who underwent RARC with ICUD from February 2018 to November 2023, performed by a single surgeon. Intraoperative and postoperative complications data were retrieved for analysis, employing our proposed mesentery-sparing technique in all cases. The primary endpoint was the incidence of intraoperative and postoperative complications directly attributable to the mesentery-sparing approach in ICUD. Secondary endpoints included other postoperative variables not directly related to mesentery preservation, such as the incidence of postoperative ileus requiring parenteral nutrition and the duration of hospitalization.

**Results:**

None of the patients experienced intraoperative or postoperative complications directly related to mesentery-sparing, such as intestinal fistulae or internal hernias. The median duration of hospitalization was 6 days, and postoperative ileus necessitating total parenteral nutrition occurred in 19% of the patients. Minor complications (Clavien–Dindo grades I-II) accounted for 27.6% of the cases and major complications (grades III-V) accounted for 20.6%.

**Conclusion:**

The mesentery-sparing technique outlined herein offers an alternative method for preserving the vascularization of intestinal segments and reducing the risk of intestinal complications in ICUD during RARC.

## INTRODUCTION

Radical cystectomy with extended pelvic lymph node dissection (EPLND) currently provides the best long-term survival and lowest local recurrence rates for the treatment of N0M0 muscle-invasive bladder cancer (1) and high-risk non-muscle-invasive bladder cancer unresponsive to Bacillus Calmette-Guerin therapy (2). However, radical cystectomy is associated with high overall morbidity and mortality rates. Although robotic techniques can reduce the morbidity of surgical wounds, intraoperative bleeding, blood transfusion rates, and the duration of hospitalization, these techniques are associated with other potential complications not directly related to the surgical approach but to the procedure itself (3, 4). In its latest retrospective review of 2,976 patients across 26 institutions from 11 countries, the International Robotic Cystectomy Consortium (IRCC) demonstrated that 60% of patients developed postoperative complications after robot-assisted radical cystectomy (RARC). Of these, 28% showed high-grade complications according to the Clavien–Dindo classification (5). The most feared complications are those arising from urinary diversion, which may be extracorporeal or intracorporeal, since these are the most dangerous, severe, and potentially fatal complications (6, 7).

The vascularization of intestinal segments, both in the excluded segment used (e.g., Bricker or neobladder) and in the distal and proximal ends of the ileum that will re-establish intestinal transit, is crucial to minimize ischemia and potential fistulas. Therefore, in this study, we present our technique that involves total preservation of the vascularization of the ileal mesentery used in urinary diversion. With this approach, we anticipate a significant reduction in intestinal complications related to the procedure.

## MATERIALS AND METHODS

Between February 2018 and November 2023, 31 patients underwent RARC with EPLND. Platinum-based neoadjuvant chemotherapy was selectively administered in some cases. This cohort included male and female patients who underwent intracorporeal urinary diversion (ICUD) with an ileal conduit or orthotopic ileal neobladder and at least 90 days of postoperative follow-up. All procedures were performed by a single experienced robotic surgeon using the da Vinci Surgical System™. Intraoperative and postoperative complications were classified according to the Clavien–Dindo criteria. This study was approved by the research ethics committee (CAAE: 67370722.9.0000.5125, n 6.222.638).

The primary endpoint was the appearance of intraoperative and postoperative complications directly related to preservation of intestinal vascularization for ICUD and the reconstruction of intestinal transit; the potential complications included ischemia, intestinal fistula, enteric anastomotic stricture, mesenteric bleeding and/or hematoma, and internal hernia. The secondary endpoints were other postoperative variables not directly related to mesenteric preservation, such as postoperative ileus requiring parenteral nutrition and the duration of hospitalization.

### Preoperative Preparation

The patients were maintained on a restricted liquid diet for 24 h before RARC. Antibiotic prophylaxis was performed by intravenous administration of ceftriaxone and metronidazole.

### SURGICAL TECHNIQUE

#### Patient Positioning and Port Placement

The patient was placed in a supine position under combined anesthesia (general anesthesia and spinal block). The patients’ legs were placed in stirrups to maintain the lithotomy position. The surgical table was tilted to a 25° Trendelenburg position (Lloyd–Davies position).

**Figure 1:**
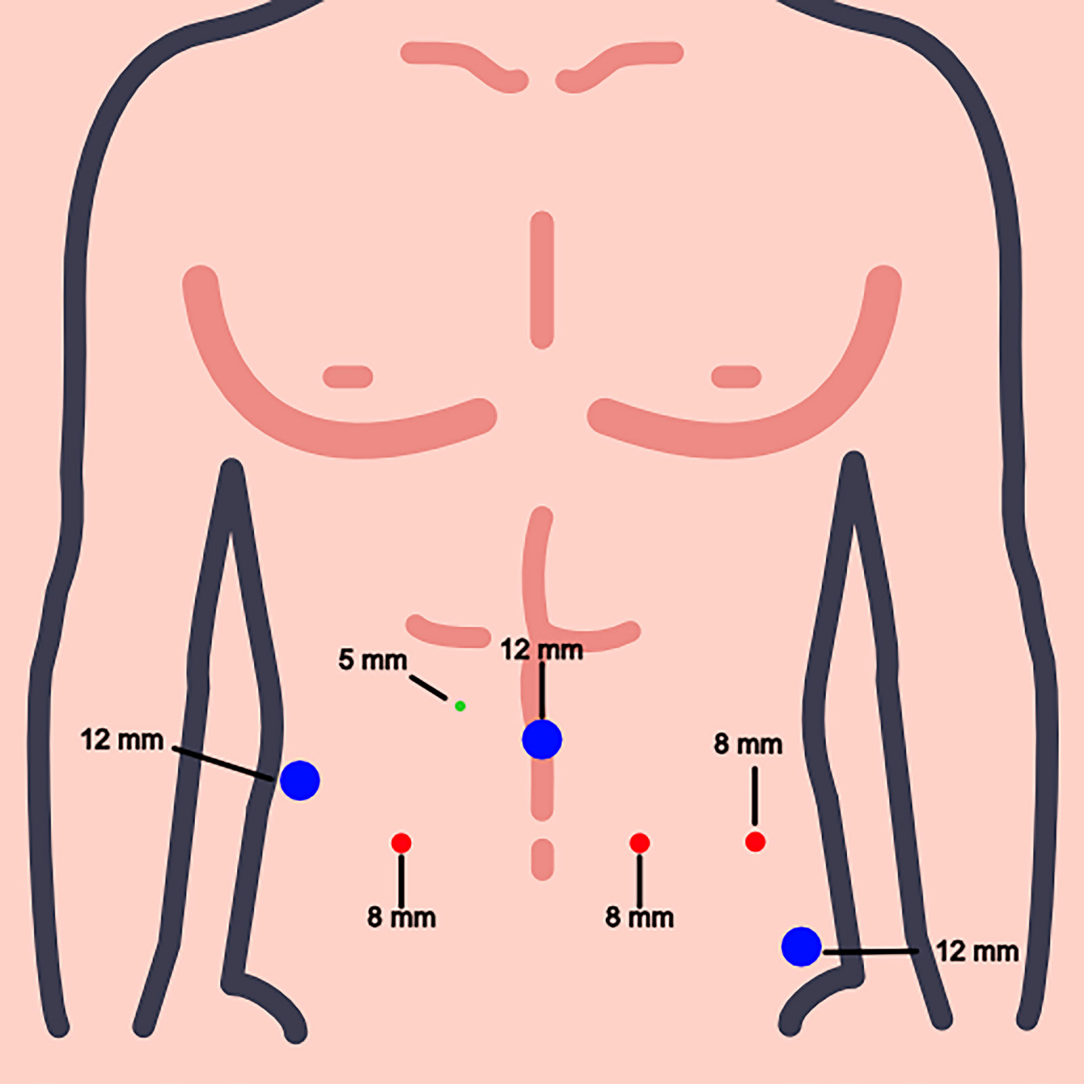
The figure shows a schematic drawing showing arrangement of trocars for radical cystectomy. Note the additional 12 mm trocar on the left side closer to the anterosuperior iliac spine.

A pneumoperitoneum of 12 mmHg was established, and a transperitoneal approach with seven ports was used. Two 12-mm trocars, one for assistance on the right side at the level of the umbilical scar and the other for assistance on the left side closer to the anterosuperior iliac spine, were used along with one 5-mm trocar for suction. Three robotic-arm trocars (8 mm) were aligned with the umbilical scar (Figure-1), and the arm that held the endoscope (8 mm or 12 mm, depending on the platform used) was placed 4 cm above the umbilical scar to facilitate ICUD. For the robotic instruments, we used Maryland bipolar forceps, monopolar scissors, Prograsper, and two needle drivers. In addition, we used 60-mm laparoscopic staplers.

#### Bladder Removal and Lymphadenectomy

Bladder removal was performed using the conventional procedure. The urethra was spared in cases involving neobladder construction. Lymphadenectomy was performed using the standard template described by Herr et al. (8).

#### Creation of an Intracorporeal Ileal Conduit with the Mesentery-Sparing Technique

**Figure 2:**
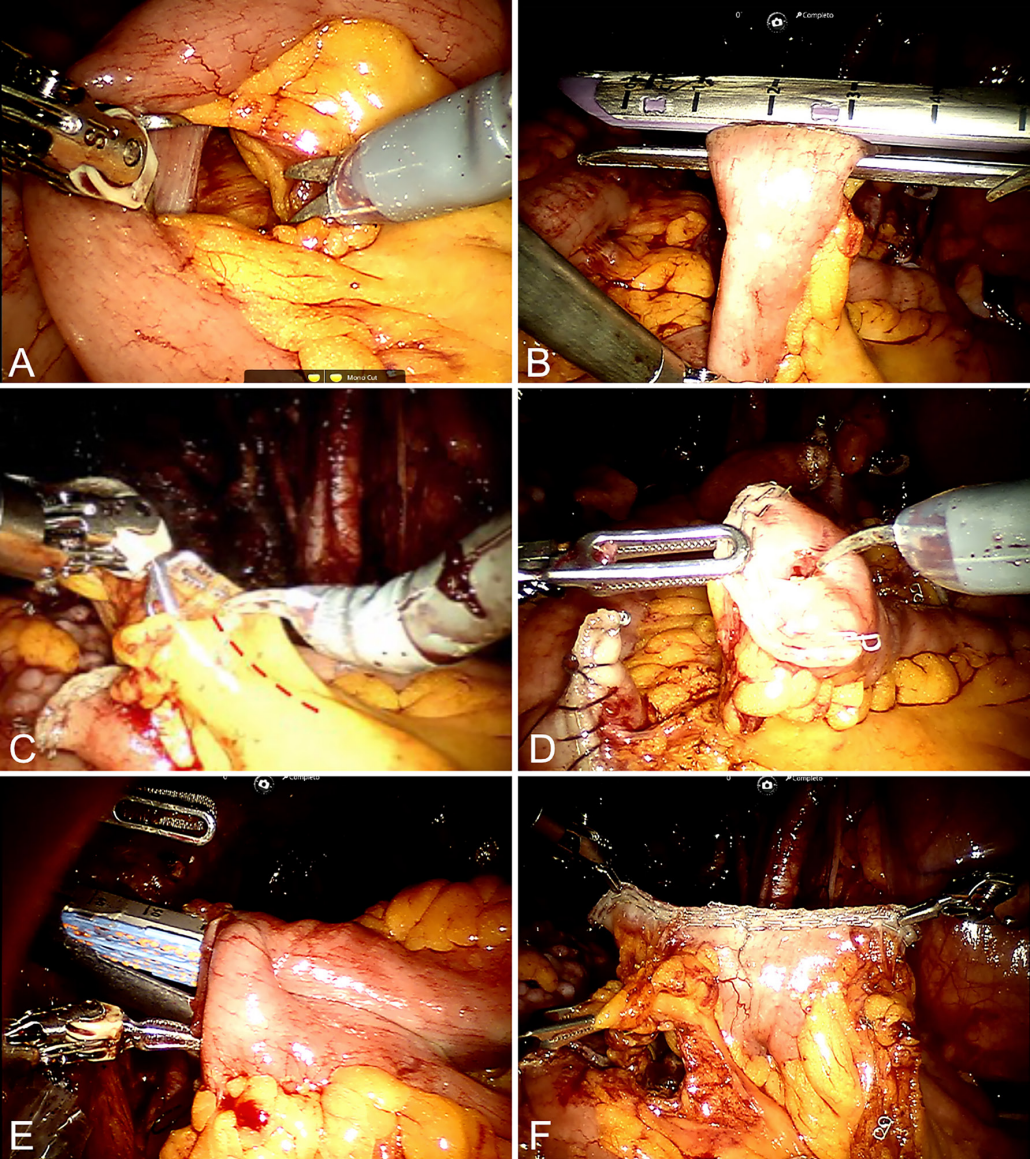
The figure shows the steps of the surgical technique.

**Figure 3:**
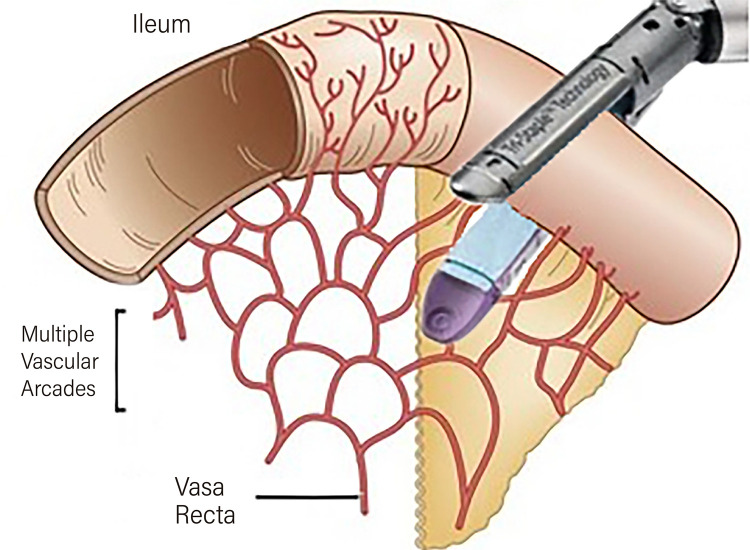
Schematic representation shows the preservation of the mesenteric vessels for the introduction of the Stapler.

A 15-cm segment of the ileum was initially demarcated 20 cm proximal to the ileocecal valve using a 20-cm silk thread with a marking knot every 5 cm to provide exact measurements. Using scissors and Maryland forceps, a small opening was made in the mesentery as close as possible to the intestinal loop wall, which was sufficient for passage of the stapler (Figure-2A). The stapler was then introduced through the right auxiliary port (12 mm) and used perpendicular to the mesentery (Figure-2B). With this maneuver, the vascularization of the entire double row of the distal ileal arcade as well as the perpendicular vessels that penetrate the intestinal wall (vasa recta) (9) was completely preserved. Our technique did not even require sectioning the arcades closest to the ileal wall, and the stapler was placed between the vasa recta (Figure-3). This technique ensured adequate blood supply to both the isolated segment of the ileal conduit and the proximal and distal ends of the ileum, which were used for reconstruction of the intestinal transit.

A) The Maryland forceps are used to dissect the space between the small bowel loop and the mesentery. B) The stapler is used at a perpendicular angle to the mesentery without advancing onto it. C) A relaxing incision is made only in the peritoneum of the mesentery, leaving it intact. D) The position of the intestinal anastomosis intuitively becomes easier to perform at the antimesenteric border. E) A new 12 mm trocar is inserted to the left for the first suture line of the entero-enteric anastomosis. F) Final aspect of the intestinal anastomosis after the reconstitution of intestinal transit.

After isolating the ileal segment, a relaxing incision of only 5 cm was made solely in the peritoneum of the mesentery, keeping the center of the mesentery, which contains the blood supply, intact (Figure-2C). Because of the perpendicular angle of the stapler to the mesentery, the exact point for enterotomy to introduce the stapler jaw for the next anastomosis was the midpoint of the staple line, which facilitated its identification for the next lateral–lateral stapling of the intestinal transit reconstruction (Figure-2D). Lateral stapling of the distal ileum (enteroanastomosis) was performed using a 12-mm auxiliary port to the left near the anterosuperior iliac spine (Figure-2E). This extra port improved the angulation of the stapler and maintained the active assistance of the fourth robotic arm. Final stapling was then performed, completing the reconstruction of the intestinal transit (Figure-2F).

#### Creation of an Orthotopic Ileal Neobladder with the Mesentery-Sparing Technique

Regardless of whether the Lavallee et al. (10) or Gaston et al. (11) technique was used, the urethra was first anastomosed with the ileal segment that became the neobladder. At this stage, a Rocco et al. suture (12) may be performed depending on the surgeon’s preference. After anastomosis with the urethra, 25–30-cm segments afferent and efferent to the urethra were selected. The same mesentery-sparing technique described above was employed for sectioning and isolating the ileal segments. Even with the ileal segment fixed to the urethral anastomosis, this technique was feasible and did not require further release from the mesentery. For neobladders, the neovesical catheter was generally removed 14–21 days after surgery.

#### Uretero-ileal Anastomosis

Uretero-ileal anastomosis was performed using continuous polygalactin sutures. Ureteral stents are placed before the ureteral anastomosis and generally left in place for approximately 15 days postoperatively. In both forms of ICUD, an intraperitoneal drain was left in place during the hospitalization period. All patients were treated postoperatively according to the Enhanced Recovery After Surgery (ERAS) protocols (13).

#### Data Collection

Data were collected retrospectively by reviewing medical records. Age, sex, body mass index (BMI), and American Society of Anesthesiology (ASA) score were used to classify the performance status and functional state of the patients. Preoperative hemoglobin level, operative time, estimated blood loss, postoperative hemoglobin level, duration of hospitalization, type of urinary diversion, postoperative complications up to 90 days based on the Clavien–Dindo classification, and pathological characteristics were recorded for each patient. Approval was granted by the ethics committees of the participating institutions.

#### Statistical Analysis

Descriptive statistics, including the mean, standard deviation, and median, were used to report continuous variables, while frequencies were reported for categorical variables. For the analysis of discrete variables, values were reported as percentages of the total number of participants in each study group. Statistical analysis was performed using SPSS® Statistics Software.

## RESULTS

In Table-1 presents the demographic characteristics of the participants. A total of 31 patients, including 25 males and six females, underwent RARC with ICUD, and the minimum postoperative follow-up period was 90 days. The average age of the participants was 65 years (range, 46–88 years), and the average BMI was 26 (range, 17–40). The ASA scores were distributed as follows: 1, 14%; 2, 75%; and 3, 11%. All patients were managed in accordance with the ERAS protocol. Diet was introduced 24hs. postoperatively and progressively advanced to a regular diet. Patients were encouraged to ambulate from 4 h postoperatively. An intracorporeal neobladder and a non-continent ileal conduit were created in 10% and 90% of the cases, respectively. The average operative times for creating the ileal conduit and neobladder were 312 min (range, 159–540 min) and 397 min (range, 314–480 min), respectively. The average blood loss was 440 mL (range, 150–1,100 mL). The preoperative hemoglobin level was 12.9 g/dL (range, 7.8 to 17.5 g/dL) and the 24-h postoperative hemoglobin level was 10.9 g/dL (range, 7.4 to 15.7 g/dL) (Table-1).

**Table 1:** Demographics, intraoperative data and tumor characteristics.

Patients, n	31
Male, n(%)	25 (80%)
Female, n(%)	6 (20%)
Age, av. (range)	65 (46-88)
BMI, av. (range)	26,8 (17-40)
**ASA, n(%)**	
I	4(14)
II	21(75)
III	3(11)
**Urinary diversion, n(%)**	
Ileal conduit	28(90)
Neobladder	3(10)
**Operative time (min), av**	
Ileal conduit	312 (159 - 540)
Neobladder	397 (314 - 480)
**Estimated blood loss, mL**	440 (150 – 1,100)
Preoperative Hb g/dL, av. (range)	12.9 (7.8 – 17.5)
Postoperative Hb g/dL, av. (range)	10.9 (7.4 – 15.7)
Length of hospital stay, (days) median	6
**Histology (%)**	
Pure urothelial carcinoma	58
Urothelial cell carcinoma with differentiation	23
No identifiable tumor in the specimen	16
Other	3
Pathological stage T	
Organ confined disease, pT0-pT2b (%)	66.6
Extravesical disease, pT3a-pT4a (%)	33.4
Lymph node count, av. (range)	19 (2 – 33)
**Pathological N, n(%)**	
pN0	21(67)
pN1	4(13)
pN2	4(13)
pN3	2(7)
**Clavien-Dindo (%)**	
No complications	51.7
Minor complications (grade I-II)	27.6
Major complications (grade III-V)	20.6

BMI = Body Mass Index; ASA = American Society of Anesthesiologists

The pathological characteristics of the cystectomy specimens were as follows: 58.1% were pure urothelial cell carcinomas, 22.6% were urothelial cell carcinomas with differentiation or variants, and 16.1% exhibited no identifiable tumors within the specimen, indicating either a favorable response to neoadjuvant treatment or complete resection during endoscopic surgery. Organ-confined tumors accounted for 66.6% of the cases (T0-T2b), and 33.4% of the cases showed tumors with extravesical local involvement (T3a-T4a). The average number of lymph nodes dissected was 19 (range, 2–33), and only one case showed positive margins due to an undifferentiated neuroendocrine tumor.

In the evaluation of the primary endpoint, none of the patients showed intraoperative mesenteric vessel bleeding or mesenteric hematoma during anastomosis inspection. No ischemia was observed during the surgery. Only four patients underwent assessments with indocyanine green, and none showed signs of poor perfusion of the intestinal segments. Over the first 90 days postoperatively, none of the patients developed an intestinal fistula, stenosis, or internal hernia related to urinary diversion. In assessments of the secondary endpoint, the median duration of hospitalization was 6 days, and postoperative ileus requiring total parenteral nutrition occurred in 19% of the patients.

Minor complications (Clavien–Dindo grades I-II) accounted for 27.6% of the cases and major complications (grades III-V) accounted for 20.6%. Two patients died: the oldest patient in the series (88 years) died due to mechanical ventilation-related pneumonia and another patient died due to aspiration pneumonia following a decrease in consciousness level caused by the medication used to treat hiccups on the day of hospital discharge (Table-1).

We can observe the video showing the steps of this technique in the link below:

**WATCH THE FULL VIDEO**

## DISCUSSION

In comparison with conventional laparoscopic techniques, the robotic system facilitates more complex reconstructions, including ICUD during radical cystectomy. Nonetheless, radical cystectomy with complete ICUD remains a technically challenging procedure. In a systematic review and meta-analysis to compare RARC with ICUD versus extracorporeal urinary diversion (ECUD) and to assess the complications, perioperative outcomes, and oncological results, the surgical time and perioperative complications, including general or major, short- or medium-term complications, were comparable between ICUD and ECUD. ICUD may potentially expedite intestinal function recovery by reducing excessive bowel manipulation and exposure to ambient air. Although a recent meta-analysis indicated a trend toward reduced paralytic ileus with ICUD, this was not substantial enough to establish a statistically significant difference.

The IRCC documented a significant increase in the use of ICUD, which has risen from 9% in 2005 to 97% in 2016. A more recent publication from the IRCC revealed that among patients registered in the database, 64% underwent ICUD with a neobladder. These data underscore the increasing adoption of ICUD, which shows a statistically significant trend (p < 0.01) (14).

In open, laparoscopic, or robotic radical cystectomy with ECUD, the classical technique requires extensive mobilization of the mesentery from the ileal segments designated as the conduit or neobladder as well as from the proximal and distal ends of the ileal loop to facilitate the reconstruction of intestinal transit. During the initial phase of ECUD in RARC procedures, substantial mesenteric mobilization is necessary to ensure that the targeted ileal segments reach the patient’s external abdominal area (15-17). This task is exceptionally challenging, and the level of difficulty is largely contingent on the length of the mesenteric root or the patient’s level of obesity. Consequently, in most instances, the procedures entail ligation of the distal arcade of ileal irrigation. This practice of distal arcade ligation has persisted even after the introduction of complete intracorporeal reconstruction. Guru, K. et al. (18), in the description of their technique, refer to the surgical phase of isolating the intestinal segment for enhanced mobilization as the “Marionette Technique,” which involves substantial opening of the mesentery prior to employing the 45-mm Endo GIA® stapler. Hosseini et al. (19) in their study, also employed advancement of the stapler across the mesentery to gain mobility and perform ligation of the initial arcade, often at the risk of edge ischemia. Similar to the descriptions provided in these two studies, this step of the procedure, which involves sectioning of the ileal loop along with the mesentery, has also been well documented in studies by Abreu and Gill (20), Fumo et al. (21), Pruthi et al. (22), Siemer et al. (23), Johnson et al. (24), and Baboudjian et al. (25).

The hallmark of our study was complete preservation of the mesentery, since ICUD requires minimal mesenteric mobility. In our technique, we abstained from ligating the blood arcades, including the most distal blood arcades (vasa recta), thereby significantly minimizing ischemia, including subclinical ischemia that is not detectable through visual inspection or the use of indocyanine green. Another advantage of our technique was the reduced risk of mesenteric bleeding and local hematoma formation. Moreover, since our technique did not create substantial peritoneal “windows”, it did not require separate allocations of time to close the peritoneum to prevent internal hernias, since the incisions are minimal.

An important point to consider is that ECUD can be performed with the “transillumination” technique to precisely identify the exact location for ligation and sectioning of distal vascular arcades. However, in intracorporeal reconstruction, this technique is usually not available, and arcade ligation is performed more imprecisely. Another aspect related to costs arises when patients have more dilated or larger-caliber ileal loops. In such cases, some surgeons opt to use an additional stapler load to advance over the mesentery, which incurs additional costs (26).

The Pasadena Consensus Panel suggested a hospital stay of 5–10 days after procedures performed by highly experienced surgeons (>100 cases). The median hospital stay in our study was 6 days, which aligns with the findings of other studies. This may have been influenced by the mesentery-sparing technique. This finding could be attributed to the [1] less intestinal loop manipulation in this technique, [2] less ischemia of the distal and proximal ends of the intestinal transit reconstruction, and [3] smaller openings or “windows” in the mesentery. While the procedures in our study were performed by an experienced surgeon, an important consideration is that the complication rate in such challenging procedures is intrinsically linked to the experience level and learning curve of the surgeon (27).

Our group initially adopted the traditional technique, which involved advancing the stapler over the mesentery to facilitate mobilization. However, after an in-depth study of the vascular anatomy of the distal ileum and the realization that mesenteric mobilization was unnecessary, we transitioned to a mesentery-preserving technique. The ileum is irrigated by an extensive network of arteries originating from the superior mesenteric artery that cross the mesentery. This network consists of multiple arterial branches, known as arterial arcades, which form from 15-18 branches and culminate in the terminal “vasa recta” or straight branches, responsible for supplying blood to the ileum. A distinctive feature of the ileum in comparison with the jejunum is the presence of a double row of arcades, which are closer to the ileal wall. In our technique, we did not section these arcades, even those closest to the ileum wall, and the stapler was positioned between the straight vessels, which were also preserved, thus avoiding ligation of these vessels.

In describing their technique, Jeglinschi et al. used indocyanine green to confirm the vascular anatomy and subsequently make a peritoneal incision (28). In our study, we used indocyanine green in four cases but did not observe local ischemia, either in the urinary diversion segments or in the proximal and distal ends of the enteroanastomosis. Although indocyanine green can help identify ischemia, examinations using this dye cannot serve as a preventive measure. If ischemia is detected, resection of the ischemic segment is necessary. Notably, neither indocyanine green nor visual inspection of the ileal wall may reveal mild reductions in irrigation, which constitute a form of subclinical ischemia (28, 29).

An alternative to minimize ischemia of the loop ends after sectioning was described by Desai et al. (26) in their study outlining the technical nuances aimed at improving the efficiency of the procedure. Regarding this particular surgical point, the authors mentioned deepening the extent of the mesenteric incision for better mobility, in addition to resecting 5 cm of the ileum proximal to the isolated loop. In techniques involving the creation of a wide peritoneal window, the opening must be closed after intestinal anastomosis to prevent the development of internal hernias. This process is time-consuming. However, in our technique, the peritoneal opening was minimal, eliminating the need to close it, simplifying the procedure, and reducing the surgical time.

Another pertinent point is the importance of using an additional 12-mm trocar on the patient’s left flank near the anterosuperior iliac spine instead of using it on the robot’s third arm with the corresponding 8-mm trocar inside. In addition to having another arm to assist in the subsequent steps, angulation to make the first intestinal anastomosis became much more favorable with this approach.

In addition, our use of the stapler ensured that the incision for the intestinal anastomosis was intuitively easier to make on the anti-mesenteric edge of the loop (Figure-2B). The principles of ileal segment selection in terms of size have been covered extensively in the literature; however, few studies have described or even addressed the issue of using a stapler over the mesentery. Some published technique videos have shown the advancement of the stapler over the mesentery. However, the Stapler Endo GIA® manual does not contain any technical reference to guide its use over the mesentery (29).

The main strength of our study is the collection and retrospective analysis of perioperative outcomes and complication rates associated with intestinal diversions. To our knowledge, this is the first report describing a mesentery-preserving technique for RARC. However, the small size of our case series, the absence of a control group that underwent mesenteric sectioning, and the relatively short follow-up period were limitations of our study. Nevertheless, all patients underwent follow-up for intestinal complications for at least 90 days, after which such complications became rarer.

In conclusion, ICUD during RARC can be safely performed within an acceptable operative time. The mesentery-sparing technique described here can be an alternative for preserving the superior vascularization of the intestinal segments in ICUD and reducing ileal mobilization.

## References

[1] Alfred Witjes J, Max Bruins H, Carrión A, Cathomas R, Compérat E, Efstathiou JA (2024). European Association of Urology Guidelines on Muscle-invasive and Metastatic Bladder Cancer: Summary of the 2023 Guidelines. Eur Urol.

[2] Babjuk M, Burger M, Capoun O, Cohen D, Compérat EM, Dominguez Escrig JL (2022). European Association of Urology Guidelines on Non-muscle-invasive Bladder Cancer (Ta, T1, and Carcinoma in Situ). Eur Urol.

[3] Sobhani S, Ghoreifi A, Douglawi A, Ahmadi H, Miranda G, Cai J (2023). Perioperative mortality for radical cystectomy in the modern Era: experience from a tertiary referral center. Int Braz J Urol.

[4] Liu H, Zhou Z, Yao H, Mao Q, Chu Y, Cui Y (2023). Robot-assisted radical cystectomy vs open radical cystectomy in patients with bladder cancer: a systematic review and meta-analysis of randomized controlled trials. World J Surg Oncol.

[5] Dindo D, Demartines N, Clavien PA (2004). Classification of surgical complications: a new proposal with evaluation in a cohort of 6336 patients and results of a survey. Ann Surg.

[6] Knap MM, Lundbeck F, Overgaard J (2004). Early and late treatment-related morbidity following radical cystectomy. Scand J Urol Nephrol.

[7] Konety BR, Allareddy V, Herr H (2006). Complications after radical cystectomy: analysis of population-based data. Urology.

[8] Herr H, Lee C, Chang S, Lerner S, Bladder Cancer Collaborative Group (2004). Standardization of radical cystectomy and pelvic lymph node dissection for bladder cancer: a collaborative group report. J Urol.

[9] Collins JT, Nguyen A, Badireddy M (2023). Treasure Island (FL): StatPearls Publishing.

[10] Lavallée E, Wiklund P (2021). The Studer Neobladder: An Established and Reproducible Technique for Intracorporeal Urinary Diversion. Eur Urol Open Sci.

[11] Gaston R, Ramírez P (2019). Intracorporeal neobladder. Arch Esp Urol.

[12] Rocco B, Assumma S, Calcagnile T, Sangalli M, Turri F, Micali S (2022). Reproducibility of a modified posterior reconstruction during robotic intracorporeal neobladder reconfiguration. Int Braz J Urol.

[13] Cerantola Y, Valerio M, Persson B, Jichlinski P, Ljungqvist O, Hubner M (2013). Guidelines for perioperative care after radical cystectomy for bladder cancer: Enhanced Recovery After Surgery (ERAS(®)) society recommendations. Clin Nutr.

[14] Dalimov Z, Iqbal U, Jing Z, Wiklund P, Kaouk J, Kim E (2022). Intracorporeal Versus Extracorporeal Neobladder After Robot-assisted Radical Cystectomy: Results From the International Robotic Cystectomy Consortium. Urology.

[15] Chan KG, Guru K, Wiklund P, Catto J, Yuh B, Novara G (2015). Robot-assisted radical cystectomy and urinary diversion: technical recommendations from the Pasadena Consensus Panel. Eur Urol.

[16] Pruthi RS, Smith A, Wallen EM (2008). Evaluating the learning curve for robot-assisted laparoscopic radical cystectomy. J Endourol.

[17] Parekh DJ, Messer J, Fitzgerald J, Ercole B, Svatek R (2013). Perioperative outcomes and oncologic efficacy from a pilot prospective randomized clinical trial of open versus robotic assisted radical cystectomy. J Urol.

[18] Guru K, Seixas-Mikelus SA, Hussain A, Blumenfeld AJ, Nyquist J, Chandrasekhar R (2010). Robot-assisted intracorporeal ileal conduit: Marionette technique and initial experience at Roswell Park Cancer Institute. Urology.

[19] Hosseini A, Adding C, Nilsson A, Jonsson MN, Wiklund NP (2011). Robotic cystectomy: surgical technique. BJU Int.

[20] Abreu SC, Gill IS (2005). Minim Invasive Uro-Oncologic Surg.

[21] Fumo MJ, Badani KK, Menon M (2008). Robotics in urologic surgery.

[22] Pruthi RS, Nix J, McRackan D, Hickerson A, Nielsen ME, Raynor M (2010). Robotic-assisted laparoscopic intracorporeal urinary diversion. Eur Urol.

[23] Siemer S, Kamradt J, Stöckle M (2011). Laparosc Robot Surg Urol Atlas Stand Proced.

[24] Johnson D, Castle E, Pruthi RS, Woods ME (2012). Robotic intracorporeal urinary diversion: ileal conduit. J Endourol.

[25] Baboudjian M, Gondran-Tellier B, Michel F, Abdallah R, Rouy M, Gaillet S (2021). Miami Pouch: A Simple Technique for Efficient Continent Cutaneous Urinary Diversion. Urology.

[26] Desai MM, de Abreu AL, Goh AC, Fairey A, Berger A, Leslie S (2014). Robotic intracorporeal urinary diversion: technical details to improve time efficiency. J Endourol.

[27] Tuderti G, Mastroianni R, Brassetti A, Bove AM, Misuraca L, Anceschi U (2021). Robot-assisted radical cystectomy with intracorporeal neobladder: impact of learning curve and long-term assessment of functional outcomes. Minerva Urol Nephrol.

[28] Jeglinschi S, Carlier M, Denimal L, Guillonneau B, Chevallier D, Tibi B (2020). Intracorporeal urinary diversion during robot-assisted radical cystectomy using indocyanine green. Can J Urol.

[29] Gaya JM, Uleri A, Sanz I, Basile G, Verri P, Hernandez P (2023). Robot-assisted radical cystectomy and ileal conduit with HugoTM RAS system: feasibility, setting and perioperative outcomes. Int Braz J Urol.

